# Bone Fracture Risk and Renal Dysfunction in a Highly Cadmium Exposed Thai Population

**Published:** 2018-08-04

**Authors:** Kowit Nambunmee, Muneko Nishijo, Witaya Swaddiwudhipong, Werawan Ruangyuttikarn

**Affiliations:** ^1^ Occupational Health and Safety, Major of Public Health, School of Health Science, Mae Fah Luang University, Chiang Rai, Thailand; ^2^ Department of Public Health, Kanazawa Medical University, Ishikawa, Japan; ^3^ Department of Community and Social Medicine, Mae Sot General Hospital, Tak, Thailand; ^4^ Department of Forensic Medicine, Faculty of Medicine, Chiang Mai University, Chiang Mai, Thailand

**Keywords:** Cadmium poisoning, Proximal renal tubular dysfunction, Calcium metabolism disorder, Thailand, Bone demineralization, Pathologic

## Abstract

**Background:** Paddy fields in the Mae Sot, Tak Province of Thailand are polluted with unsafe levels of cadmium (Cd). Elderly populations have a high Cd body burden, putting them at elevated risk of renal dysfunction and bone fractures. We aimed to compare bone fracture risk between glomerular dysfunction, proximal tubular dysfunction, and calcium (Ca) handling abnormalities.

**Study design:** A cross-sectional study.

**Methods:** Serum osteocalcin and cross-linked N-telopeptide of type I collagen were used to detect bone metabolism abnormalities, whereas glomerular filtration rate, serum cystatin C, urinary β2-microglobulin (β2-MG) and fractional excretion of calcium (FECa) were used to indicate renal dysfunction. Urinary Cd was used as an exposure marker.

**Results:** FECa >2% was associated with high bone fracture risk in both genders. The adjusted odds of bone fracture risk were 6.029 and 3.288 in men and women, respectively with FECa >2% relative to the FECa <2% group. Proximal tubular dysfunction and glomerular dysfunction did not significantly relate to the risk of bone fracture.

**Conclusions:** Abnormal Ca handling is a key risk factor for bone fracture in Cd-exposed people. Men and women were at risk of bone fracture risk at a similar rate. FECa was a specific indicator of Ca wasting and was more cost-effective compared to β2-MG and serum cystatin C. We recommend using FECa to monitor abnormal Ca metabolism in individuals with FECa>2%. Reduced renal toxicant exposure and Ca supplementation are recommended for Cd-exposed populations to reduce bone fracture risk.

## Introduction

High levels of cadmium (Cd) are exposure causes renal and bone abnormalities. Itai-itai disease is the most severe pathology caused by high-level cadmium exposure^1^. At low Cd exposure levels, exposed populations are also at risk of bone disease. The prevalence of osteoporosis in a Chinese Cd-exposed population was 2.09 times higher than reference group^2^, and bone fracture risk in an exposed population from Sweden was 8.80 times higher than a reference population^3^.

Cadmium contamination was identified in the Mae Sot district in Thailand, where a zinc mine operated. A survey of Cd levels in rice paddy soil showed that Cd in the polluted area ranged between 0.5-284 mg/kg, which was higher than in other areas of Thailand (0.01-1.30 mg/kg)^4^. In addition, 95% of surveyed rice grain samples were contaminated with Cd levels greater than 0.1 mg/kg, the level considered safe^5^. Rice is a staple food for the population of Mae Sot, putting them at increased risk of Cd toxicity^6^. There were 7697 inhabitants in the contaminated area and 7.2% of those inhabitants showed urinary Cd (U-Cd) >5 µg/g Cr^6^, a level associated with increased proximal tubular dysfunction^7^. A follow-up survey in 2007 found a 19.9% prevalence of permanent proximal tubular dysfunction and a 16.9% prevalence of glomerular dysfunction among the Cd-exposed population, which was higher than an unexposed Thai population^8^. Their exposure level was also greater than the threshold level for osteoporosis and increased bone fracture risk^9^.

Biomarkers of bone metabolism status have the advantage of identifying changes in bone metabolism and physiology earlier than measurements of bone mineral density^10^. Bone biomarker determination is a non-invasive and comparatively inexpensive tool to assess metabolic bone disease. Serum osteocalcin (OC) and urine cross-linked N-telopeptide of type I collagen (NTx) levels have previously been associated with an increased risk of bone fracture in clinical and epidemiological studies^11^. In a Cd-polluted area in Japan, associations between U-Cd and NTx and Cd-induced osteoporosis gradually developed in exposed subjects after cessation of exposure^12^. A proposed explanation for Cd-induced bone disease is kidney dysfunction, particularly proximal tubular abnormalities^13^. However, evidence for a direct relationship between Cd exposure and decreased bone mass in the absence of tubular dysfunction has also been observed^14^.

To preserve bone health and provide suitable health promotion programs in Cd-exposed populations, the relationship between kidney dysfunction and bone metabolism needs to be elucidated. We aimed to quantify the association between renal abnormality and bone fracture risk in a Cd-exposed population from Thailand using biomarkers.

## Methods

### Population and biological sampling

Study participants from Mae Sot, aged >50 yr with urinary cadmium levels quantified as higher than the reference level 5 µg/g Cr in a 2004 survey were enrolled^6^. The target sample size was 554 subjects, and of these 554 subjects, 419 (75.63 %) agreed to participate including 158 men and 261 women.

Informed consent was obtained from all participants. The study protocol was conducted in accordance with the Declaration of Helsinki as described in our previous report^15^.

Urine samples were collected in polyethylene bottles after the subjects underwent a physical examination and anthropometric measurements. Each urine sample was divided into three (3–5 ml) aliquots. In samples with pH <5, the pH of one of the three aliquots was adjusted to pH 6–8 by 0.5 N sodium hydroxide to prevent the degradation of β_2_-microglobulin in acidic conditions. A trained nurse drew five-to-ten milliliters of venipuncture blood. All aliquots were then frozen and stored at −20 °C until analysis.

### Urinary cadmium measurement

Urinary cadmium concentrations were quantified using a flameless atomic-absorption spectrometer (Shimadzu Model AAS-6300, Japan), with palladium chloride in 5% nitric acid solution as a modifier. Method validation of the analytical techniques was performed and verified by certified standard reference materials (The National Institute of Standards and Technology, Washington, DC, USA)^16^. Urinary creatinine concentrations were measured by a method based on the Jaffe reaction.

### Serum and urinary calcium measurements

Serum and urinary calcium were quantified by a colorimetric assay using an automated analyzer (Coulter HmX, Konelab 30 and Bechman Synchron CX3) at Mae Sot General Hospital. The laboratory was evaluated and certified by the Bureau of Laboratory Quality Standards, Ministry of Public Health, Thailand. The fractional excretion of calcium (FECa) was calculated based on the serum and urinary calcium concentrations ^17^.

### Renal and bone markers determination

The concentration of β_2_-microglobulin (β_2_-MG) in urine was determined by enzyme immunoassay (GLAZYME β_2_microglobulin-EIA test kit, Sanyo Chemical Industries, Ltd., Japan). Serum cystatin C concentrations (Cystatin C) were determined by a latex particle-enhanced turbidimetric immunoassay PET kit (Dako, Glostrup, Denmark). Serum osteocalcin (OC) was measured by immunoassay. Urinary type I collagen crosslinked N-telopeptide (NTx), was measured by a competitive enzyme immunoassay ^15^. OC and NTx were used as biomarkers of bone turnover^15^.

Estimation of glomerular filtration rate (GFR) was calculated from serum creatinine using the MDRD equation. GFR <60 ml/min/1.73 m^2^ defines chronic kidney disease^18^. Cystatin C levels >1.4 mg/L indicate glomerular dysfunction^19^, β_2_-MG levels >1000 µg/g Cr show irreversible proximal tubular dysfunction^20^, and FECa levels >2% indicate abnormal high excretion of Ca via urine^21^.

Bone fracture risk was identified by a high NTx level. The cut-off values to indicate bone fracture risk were >66.2 nmol BCE/mmol Cr in men, and >89.0 nmol BCE/mmol Cr in women^11^.

### Data analysis

U-Cd, β_2_-MG, and NTx were log-transformed to correct for departures from normal distributions. Bivariate associations were calculated using partial correlations, controlling for age, and visualized using scatter plots. Mean comparisons of biomarkers between genders were performed by ANCOVA adjusting for age. The Chi-square test was used to determine the distribution of subjects at high bone fracture risk according to categorized renal biomarker concentrations. Adjusted odds ratios were calculated by logistic regression. In the logistic regression model, the dependent variable was bone fracture risk indicated by high NTx level whereas the independent variables were age, BMI, smoking status (0=nonsmoker, 1=smoker), urinary cadmium, GFR, Cystatin C, β_2_-MG, and FECa. *P*-values of 0.05 or less were considered statistically significant.

## Results

The mean age was 64.56 yr in men and 61.52 yr in women. After adjustment by mean age, the mean Cd exposure level was 6.81 µg/g Cr in men and 7.29 µg/g Cr in women, which was not significant difference between genders (Table 1). The β_2_-MG mean concentration in men was higher than women were (765.60 vs 250.03 µg/g Cr, *P*<0.001). The Cystatin C mean concentration in men was borderline significantly higher than women were (1.36 vs 1.27, *P*=0.051). OC and NTx mean in women were significantly higher than men were.

**Table 1:** Comparison of BMI, urinary cadmium, renal dysfunction markers and bone turnover markers between men and women, after adjusting for age

**Variables**	**Men,** **n=158**		**Women, n=261**		**ANCOVA**
	**Mean**	**SE**	**Mean**	**SE**	***P *** **value**
Body mass index (kg/m^2^)	20.51	0.29	21.44	0.23	0.013
Urinary Cadmium (µg/g Cr)	6.81	1.05	7.29	1.04	0.291
Glomerular filtration rate	65.00	1.25	63.14	0.97	0.245
Serum cystatin C (mg/l)	1.36	0.03	1.27	0.03	0.051
Urinary β_2_-microglobulin (µg/g Cr)	765.60	1.17	250.03	1.13	0.001
Fractional excretion of calcium (%)	1.19	0.15	1.28	0.12	0.659
Serum osteocalcin (ng/ml)	5.55	0.28	6.83	0.22	0.001
NTx (nmol BCE/mmol Cr)	43.65	1.05	65.01	1.04	0.001

NTx: Urinary type I collagen cross-linked N-telopeptide

**Figure 1:**
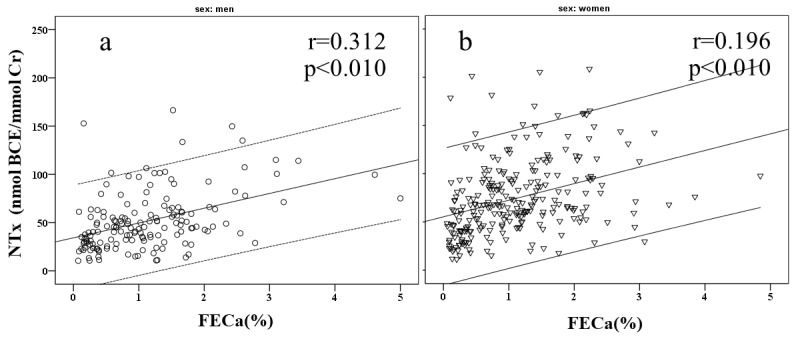
The association between Urinary type I collagen cross-linked N-telopeptide (NTx) and Fractional Excretion of calcium (FECa) in men (a) and women (b)

**Figure 2:**
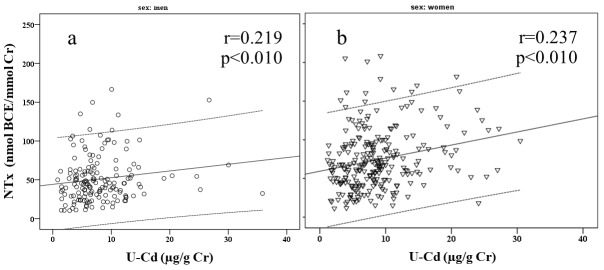
The association between Urinary type I collagen cross-linked N-telopeptide (NTx) and Urinary cadmium (U-Cd) in men (a) and women (b)

Partial correlations between OC and NTx and U-Cd, GFR, Cystatin C, and FECa are presented in Table 2. In men, OC was significantly correlated with BMI, GFR, Cystatin C, and β_2_-MG. NTx was significantly correlated with BMI, U-Cd, GFR, β_2_-MG, and FECa. In women, OC was significantly correlated with BMI, U-Cd, GFR, Cystatin C, β_2_-MG, and FECa. NTx was significantly correlated with BMI, U-Cd, GFR, β_2_-MG, and FECa. Scatter plots highlight significant positive correlations between NTx and U-Cd (Figure 1) and NTx and FECa (Figure 2) in both genders.

**Table 2:** Partial correlations of bone markers and body mass index, urinary cadmium, and renal markers controlling for age

**Variables**	**Men**				**Women**			
	**OC**	***P*** ** value**	**NTx**	***P*** ** value**	**OC**	***P*** ** value**	**NTx**	***P*** ** value**
Body mass index (kg/m^2^)	-0.165	0.039	-0.314	0.001	-0.227	0.001	-0.325	0.001
Urinary Cadmium (µg/g Cr)	0.111	0.168	0.219	0.006	0.144	0.020	0.237	0.001
Glomerular filtration rate	-0.220	0.006	0.186	0.019	-0.183	0.003	0.254	0.001
Serum cystatin C (mg/l)	0.313	0.001	-0.001	0.988	0.290	0.001	-0.064	0.307
Urinary β_2_-microglobulin (µg/g Cr)	0.251	0.001	0.358	0.001	0.278	0.001	0.163	0.008
Fractional excretion of calcium (%)	0.097	0.226	0.312	0.001	0.215	0.001	0.196	0.001

In men, the prevalence of high bone fracture risk in individuals with β_2_-MG >1000 µg/g Cr was significantly higher compared to individuals with β_2_-MG <1000 µg/g Cr (29.73% vs 13.10%, *P*=0.010) (Table 3). Additionally, the prevalence of high bone fracture risk in men with FECa >2% was significantly elevated relative to those with FECa<2% (54.55 vs 15.44%, *P*<0.001) (Table 3). The prevalence of high bone fracture risk was significantly higher in women with FECa>2% relative to those with FECa<2% (56.10% vs 24.55%, *P* <0.001).

**Table 3:** Percentage distribution of subjects according to bone fracture risk and renal markers in men and women

**Variables**	**Men**					**Women**				
	**Negative**		**Positive**		**x2**	**Negative**		**Positive**		**x2**
	**Number**	**Percent**	**Number**	**Percent**	***P *** **value**	**Number**	**Percent**	**Number**	**Percent**	***P*** ** value**
Glomerular filtration rate (ml/min/1.73 m3 body surface area)										0.024
>60	79	75.96	25	24.04	0.176	104	65.41	55	34.59	
<60	46	85.19	8	14.81		80	78.43	22	21.57	
Cystatin C (ng/ml)										0.616
<1.4	84	79.25	22	20.75	1.000	144	69.57	63	30.43	
>1.4	41	78.85	11	21.15		40	74.07	14	25.93	
Urinary β_2_-microglobulin (µg/g Cr)										0.397
<1,000	73	86.9	11	13.10	0.011	150	71.77	59	28.23	
>1,000	52	70.27	22	29.73		34	65.38	18	34.62	
Fractional excretion of calcium (%)										0.001
<2	115	84.56	21	15.44	0.001	166	75.45	54	24.55	
>2	10	45.45	12	54.55		18	43.90	23	56.10	

High bone fracture risk (Positive) identified in: men with NTx >66.2 nmol BCE/mmol Cr; women with NTx>54.3 nmol BCE/mmol Cr

We used logistic regression models, stratified by gender, to quantify the association between kidney dysfunction and high bone fracture risk. The dependent variable in the models was bone fracture risk indicated by excess NTx (>66.2 nmol BCE/mmol Cr in men, and >89.0 nmol BCE/mmol Cr in women), while age, BMI, U-Cd, GFR, Cystatin C, β_2_-MG, and FECa were modeled as the independent variables. Men and women with FECa>2% had odds of high bone fracture risk 6.03 and 3.29 times higher respectively, (Table 4) compared to gender-matched subjects with FECa<2%.

**Table 4:** Logistic regression analysis to determine the effect of renal injury on bone fracture risk after adjusting for age, body mass index, smoking status, and urinary cadmium

** **	**Men**			**Women**		
	**Adj. Odds**	**SE**	***P*** ** value**	**Adj. Odds**	**SE**	***P*** ** value**
Age (yr)	1.034	0.028	0.232	0.966	0.020	0.073
Body mass index (kg/m^2^)	0.811	0.091	0.021	0.867	0.041	0.001
Smoking	3.390	0.499	0.014	1.592	0.317	0.143
U-Cd	6.536	0.871	0.031	3.875	0.578	0.019
GFR <60 ml/min/1.73 m2	0.426	0.564	0.130	0.695	0.322	0.258
Age (yr)	1.016	0.026	0.537	0.954	0.020	0.020
Body mass index (kg/m^2^)	0.806	0.091	0.017	0.856	0.042	0.001
Smoking	3.611	0.496	0.010	1.618	0.315	0.127
Urinary Cadmium (µg/g Cr)	6.270	0.861	0.033	3.938	0.577	0.018
Cystatin C >1.4 mg/l	0.828	0.501	0.707	1.337	0.412	0.482
Age (yr)	1.008	0.024	0.749	0.955	0.019	0.016
Body mass index (kg/m^2^)	0.811	0.091	0.022	0.860	0.041	0.001
Smoking	3.679	0.502	0.010	1.639	0.316	0.118
Urinary Cadmium (µg/g Cr)	5.089	0.881	0.065	3.768	0.581	0.022
Urinary β_2_-microglobulin >1,000 µg/g Cr	2.215	0.444	0.073	1.436	0.362	0.317
Age (yr)	1.000	0.025	0.999	0.961	0.019	0.038
Body mass index (kg/m^2^)	0.812	0.094	0.026	0.868	0.041	0.001
Smoking	2.914	0.515	0.038	1.559	0.323	0.169
Urinary Cadmium (µg/g Cr)	7.653	0.913	0.026	3.429	0.585	0.035
Fractional excretion of calcium >2%	6.029	0.544	<0.001	3.288	0.377	0.002

## Discussion

Cadmium contamination in the Mae Sot region of Thailand has been reported since 2001^22^, and the exposed population has a high prevalence of proximal tubular dysfunction^5^. Cd disturbs bone metabolism via enhanced proximal tubular dysfunction^13^. An increased prevalence of osteoporosis in Cd-exposed populations with kidney dysfunction has been reported^23^, however, glomerular dysfunction showed no association with reduced bone mass^24^. The proposed mechanisms of Cd osteotoxicity included Cd accelerated bone resorption^12^, inhibited incorporation of Ca ions into bone tissue, reduced Ca reabsorption from GI tract^25^, enhanced wasting of Ca into urine^26^, and decreased production of the active vitamin D metabolite, 1α,25(OH)_2_D^27^.

In this study, we used published biomarker reference levels to define renal pathology and bone metabolism imbalance. Glomerular dysfunction was indicated by GFR <60 ml/min/1.73 m^2^ body surface and cystatin C >1.4 mg/L. Cystatin C is recognized as a more accurate marker of glomerular dysfunction than serum creatinine^28^. Proximal tubular dysfunction was indicated by β_2_-MG >1000 µg/g Cr, a concentration that reflects irreversible proximal tubular dysfunction^20^. Ca wasting was indicated by FECa >2 %^21^. Urinary NTx, a bone resorption marker, is measured to show the risk of bone fracture risk (cut-off value; men >66.2, and women >89.0 nmol BCE/mmol Cr).

Glomerular dysfunction and bone metabolism abnormalities were observed in chronic kidney disease patients. Specifically, reduced bone mass was associated with reduced glomerular filtration rate^21^. We identified a significant correlation between GFR and OC and NTx in both men and women (Table 2), indicating an association between glomerular dysfunction and bone remodeling. Serum cystatin C was also significantly correlated with OC in both genders. However, after classifying subjects according to bone fracture risk group and GFR or Cystatin C groups, no significant relationship between bone fracture risk and glomerular dysfunction was identified for either gender (Table 4). This result was in accordance with the report that the prevalence of osteoporosis did not relate to glomerular dysfunction^29^.

The proximal tubule is an accumulation site of Cd and a key target site for Cd toxicity^30^. Proximal tubular dysfunction occurs at an early stage of Cd intoxication^31^. β_2_-MG is a low molecular weight protein reabsorbed by the proximal tubules. An elevated urinary β_2_-MG indicates proximal tubular dysfunction. β_2_-MG has been recommended as a sensitive marker for Cd toxicity^32^. We found a positive correlation between β_2_-MG, OC, and NTx in both genders (Table 2). However, in logistic regression models, irreversible proximal tubular dysfunction did not relate to bone fracture risk (Table 4).

FECa levels greater than 2% indicate an impaired ability of the kidneys to reabsorb calcium back into the blood, which increases calcium wasting in the urine^16^. Increased calcium wasting was previously proposed as a sign of tubular damage and bone metabolism defects^33^. Subjects with increased calcium wasting have a higher risk of osteoporosis^34^. Calcium wasting into urine decreases serum calcium levels, causing the body to increase bone resorption to raise serum calcium^35^. Figure 2 shows a positive correlation between FECa and NTx, a bone resorption marker, which supports this assumption. High levels of bone resorption are normally balanced by increasing bone formation^35^, leading to high circulating levels of bone remodeling biomarkers. Here, we found a positive correlation between OC and FECa (Table 2). This result agrees with the finding that Itai-itai patients who showed increased Ca wasting and high rates of bone formation had low bone mineralization^1^.

When we quantified the relationship between biomarkers of kidney dysfunction and bone markers, FECa was a significant explanatory factor of bone fracture risk in both men and women (Table 4). FECa >2% increased bone fracture risk 6.03-fold in men and 3.29-fold in women. In contrast, GFR <60 ml/min/1.73m^2^, Cystatin C >1.4 mg/l, and β_2_-MG >1000 µg/g Cr showed no significant relation to bone fracture risk. Ca wasting contributes to bone fracture risk more than proximal tubular dysfunction and glomerular dysfunction.

The presence of Ca wasting is a key risk factor for Cd-induced abnormal bone remodeling. Serum Ca and urinary Ca are commonly used to determine the kidneys Ca control. However, serum Ca is normally tightly regulated in narrow range and urinary Ca is easily affected by dietary Ca^17^. Thus, serum and urinary Ca do not specifically indicate the Ca controlling function of kidney. FECa has significant advantages over both of these methods. FECa is used to indicate the percentage of Ca filtered at the glomerulus not reabsorbed in the tubules, a marker unaffected by dietary Ca. FECa has been proposed as a marker to indicate the early stages of renal dysfunction^36^ and is also inexpensive to measure relative to Cystatin C and β2-MG. A linear relationship between FECa and reduced bone mass, provided further evidence for the utility of FECa as a biomarker in this context^36^.

Women were previously proposed to be at higher risk for Cd osteotoxicity than men^37^ but in this study men with high FECa had a bone fracture risk odds of 6.03 compared to low FECa men (*P* <0.001, Table 4). Evidence supporting a relationship between Cd exposure and elevated bone toxicity in men^38^. Male rats with heavy Cd exposure showed low levels of bone mineralization, decreased bone mass, and high levels of bone remodeling markers relative to non-exposed rats^25^. Men exposed to low levels of Cd also showed an inverse correlation between Cd exposure and bone mass^39^. Therefore, the bone health of Cd-exposed men should be monitored in addition to that of women.

## Conclusion

The half-life of Cd in the human body is more than 10 years^25^. Cd related pathology appears in the elderly when the body cannot handle Cd toxicity. Since the Cd, contamination in the Mae Sot region of Thailand has not been fully remediated and further exposure continues, the inhabitants in this area continue to be at elevated risk of bone disease. Here, we found high bone fracture risk in study participants who showed FECa>2%. Calcium wasting was a dominant explanatory factor of bone fracture risk, showing the utility of monitoring this biomarker in Cd-exposed populations. Calcium supplements, renal toxicant exposure reduction, and regular health check-ups are essential preventive measures to reduce bone fracture risk in Cd-exposed populations. We recommended the use of FECa as an indicator of calcium wasting in Cd-exposed populations.

## Acknowledgements

We would like to thank Asst. Prof. Dr. Justin Colacino for his kind of proofreading.

This research was supported by Kanazawa Medical University, Japan and Mae Fah Luang University, Thailand.

## Conflict of interest statement

The authors declare that there is no conflict of interests.

## Funding

Department of Public Health, Kanazawa Medical University, Japan.

## Highlights

- Calcium wasting was a cause of bone fracture risk in a cadmium-exposed population.
- Permanent proximal tubular dysfunction showed no relation to bone fracture risk.
- Fractional excretion of calcium was a good marker for the determination of calcium wasting.

## References

[1] Nogawa K, Suwazono Y. Itai-Itai Disease. In: Jerome ON, ed. Encyclopedia of Environmental Health. Burlington: Elsevier; 2011.

[2] Wang H, Zhu G, Shi Y, Weng S, Jin T, Kong Q (2003). Influence of environmental cadmium exposure on forearm bone density. J Bone Min Res.

[3] Järup Alfvén TL (2004). Low level cadmium exposure, renal and bone effects - The OSCAR study. Bio Metals.

[4] Zarcinas B, Pongsakul P, McLaughlin M, Cozens G (2004). Heavy metals in soils and crops in Southeast Asia 2 Thailand. Environ Geochem Health.

[5] Teeyakasem W, Nishijo M, Honda R, Satarug S, Swaddiwudhipong W, Ruangyuttikarn W (2007). Monitoring of cadmium toxicity in a Thai population with high-level environmental exposure. Toxicol Lett.

[6] Swaddiwudhipong W, Limpatanachote P, Mahasakpan P, Krintratun S, Padungtod C (2007). Cadmium-exposed population in Mae Sot District, Tak Province: 1: prevalence of high urinary cadmium levels in the adults. J Med Assoc Thai.

[7] Järup Hellström L, Alfvén T, Carlsson MD, Grubb A, Persson B, Pettersson C (2000). Low level exposure to cadmium and early kidney damage: The OSCAR study. Occup Environ Med.

[8] Limpatanachote P (2007). Prevalence of renal dysfunction among persons with high urinary cadmium, Mae Sot district, Tak province. Sawanpracharak Med J.

[9] Chen X, Gan C, Zhu G, Jin T (2013). Benchmark dose for estimation of cadmium reference level for osteoporosis in a Chinese female population. Food Chem Toxicol.

[10] Garnero P (2000). Markers of bone turnover for monitoring antiresorptive treatment of osteoporosis. J für Menopause.

[11] Nishizawa Y, Nakamura T, Ohta H (2005). Guidelines for the use of biochemical markers of bone turnover in osteoporosis (2004). J Bone Miner Metab.

[12] Omote S, Kido T, Nishijo M (2006). Urinary type I collagen cross-linked N-telopeptides in inhabitants 18 years after cessation of exposure to cadmium in Japan. Bull Environ Contam Toxicol.

[13] Horiguchi H, Oguma E, Sasaki S, Miyamoto K, Ikeda Y, MacHida M (2005). Environmental exposure to cadmium at a level insufficient to induce renal tubular dysfunction does not affect bone density among female Japanese farmers. Environ Res.

[14] Nawrot T, Geusens P, Nulens TS, Nemery B (2010). Occupational cadmium exposure and calcium excretion, bone density, and osteoporosis in men. J Bone Miner Res.

[15] Nishijo M, Nambunmee K, Suvagandha D, Swaddiwudhipong W, Ruangyuttikarn W, Nishino Y (2017). Gender-specific impact of cadmium exposure on bone metabolism in older people living in a cadmium-polluted area in Thailand. Int J Environ Res Public Health.

[16] Nambunmee K, Honda R, Nishijo M, Swaddiwudhipong W, Nakagawa H, Ruangyuttikarn W (2010). Bone resorption acceleration and calcium reabsorption impairment in a Thai population with high cadmium exposure. Toxicol Mech Methods.

[17] Kido T, Nogawa K, Hochi Y (1993). The renal handling of calcium and phosphorus in environmental cadmium-exposed subjects with renal dysfunction. J Appl Toxicol.

[18] K/DOQI clinical practice guidelines for chronic kidney disease: evaluation, classification, and stratification (2002). Part 5 Evaluation of laboratory measurements for clinical assessment of kidney disease. Am J Kidney Dis.

[19] Roos JF, Doust J, Tett SE, Kirkpatrick CMJ (2007). Diagnostic accuracy of cystatin C compared to serum creatinine for the estimation of renal dysfunction in adults and children--A meta-analysis. Clin Biochem.

[20] Kido T, Nogawa K (1993). Dose-response relationship between total cadmium intake and β2-microglobulinuria using logistic regression analysis. Toxicol Lett.

[21] Borchhardt K, Sulzbacher I, Benesch T, Födinger M, Sunder-Plassmann G, Haas M (2007). Low-Turnover Bone Disease in Hypercalcemic Hyperparathyroidism After Kidney Transplantation. Am J Transplant.

[22] Simmons RW, Pongsakul P, Saiyasitpanich D, Klinphoklap S (2005). Elevated Levels of Cadmium and Zinc in Paddy Soils and Elevated Levels of Cadmium in Rice Grain Downstream of a Zinc Mineralized Area in Thailand: Implications for Public Health. Environ Geochem Health.

[23] Chen X, Zhu G, Jin T, Lei L, Liang Y (2011). Bone mineral density is related with previous renal dysfunction caused by cadmium exposure. Environ Toxicol Pharmacol.

[24] Jin T, Wu X, Tang Y, Nordberg M, Bernard A, Ye T (2004). Environmental epidemiological study and estimation of benchmark dose for renal dysfunction in a cadmium-polluted area in China. BioMetals.

[25] Nambunmee K (2014). Osteotoxicity in the Inhabitants of a Cadmium Polluted Area. J Health Res.

[26] Kazantzis G (2004). Cadmium, osteoporosis and calcium metabolism. BioMetals.

[27] Nogawa K, Kobayashi E, Yamada Y, Honda R, Teruhiko K, Ikiko T (1984). Parathyroid hormone concentration in the serum of people with cadmium-induced renal damage. Int Arch Occup Environ Health.

[28] Madero M, Sarnak MJ, Stevens LA (2006). Serum cystatin C as a marker of glomerular filtration rate. Curr Opin Nephrol Hypertens.

[29] Jin T, Nordberg G, Ye T, Bo M, Wang H, Zhu G (2004). Osteoporosis and renal dysfunction in a general population exposed to cadmium in China. Environ Res.

[30] Leffler PE, Jin T, Nordberg GF (2000). Differential calcium transport disturbances in renal membrane vesicles after cadmium-metallothionein injection in rats. Toxicology.

[31] Trzcinka-Ochocka M, Jakubowski M, Szymczak W, Janasik B, Brodzka R (2010). The effects of low environmental cadmium exposure on bone density. Environ Res.

[32] Kido T, Nordberg G. Cadmium-induced renal effects in the general environment. In: De Broe ME, Porter GA, Bennett WM, Verpooten GA, editors. Clinical Nephrotoxins. Dordrecht: Kluwer Academic Publishers; 1998. pp. 345-61.

[33] Wallin M, Sallsten G, Fabricius-Lagging E, Ohrn C, Lundh T, Barregard L (2013). Kidney cadmium levels and associations with urinary calcium and bone mineral density: a cross-sectional study in Sweden. Environ Health.

[34] Åkesson A, Bjellerup P, Lundh T (2006). Cadmium-induced effects on bone in a population-based study of women. Env Health Perspect.

[35] Schutte R, Nawrot TS, Richart T, Thijs L, Vanderschueren D, Kuznetsova T (2008). Bone resorption and environmental exposure to cadmium in women: a population study. Environ Health Perspect.

[36] Nambunmee K, Swaddiwudhipong W, Ruangyuttikarn W (2016). Fractional excretion of calcium, a sensitive marker for calcium wasting in cadmium-exposed women. Toxicol Environ Health Sci.

[37] Nishijo M, Satarug S, Honda R, Tsuritani I, Aoshima K (2004). The gender differences in health effects of environmental cadmium exposure and potential mechanisms. Mol Cell Biochem.

[38] Brzóska MM, Majewska K, Kupraszewicz E (2010). Effects of low, moderate and relatively high chronic exposure to cadmium on long bones susceptibility to fractures in male rats. Environ Toxicol Pharmacol.

[39] Burm E, Ha M, Kwon H-J (2015). Association between blood cadmium level and bone mineral density reduction modified by renal function in young and middle-aged men. J Trace Elements Med Biol.

